# Schistosomiasis in School Age Children in Sierra Leone After 6 Years of Mass Drug Administration With Praziquantel

**DOI:** 10.3389/fpubh.2019.00001

**Published:** 2019-02-12

**Authors:** Yakuba M. Bah, Jusufu Paye, Mohamed S. Bah, Abdulai Conteh, Sam Saffa, Alie Tia, Mustapha Sonnie, Amy Veinoglou, Mary H. Hodges, Yaobi Zhang

**Affiliations:** ^1^Neglected Tropical Disease Program, Ministry of Health and Sanitation, Freetown, Sierra Leone; ^2^Helen Keller International, Freetown, Sierra Leone; ^3^Headquarters, Helen Keller International, New York, NY, United States; ^4^Regional Office for Africa, Helen Keller International, Dakar, Senegal

**Keywords:** schistosomiasis, neglected tropical diseases, Sierra Leone, mass drug administration, praziquantel

## Abstract

Historic data and baseline surveys showed schistosomiasis as highly/moderately endemic in 7 of 14 districts in Sierra Leone, justifying annual/biennial mass drug administration (MDA) with praziquantel. MDA commenced in 2009 and reported treatment coverage had been above the World Health Organization recommended 75% of target population. Assessment in 2012 showed significant reduction in infection. In 2016, another national school-based survey was conducted to evaluate the progress. Two schools from each category (high, moderate or low) of endemic communities in each MDA district and five schools in non-MDA districts were selected. Fifty children (25 boys and 25 girls) aged 9–14 years were randomly selected per school. Parasitological examination of 1,980 stool and 1,382 urine samples were conducted. Overall *Schistosoma mansoni* prevalence in the seven MDA districts decreased to 20.4% (95% CI: 18.7–22.3%) in 2016 from 42.2% (95% CI: 39.8–44.5%) at baseline (*p* < 0.0001). Mean overall *S. mansoni* intensity of infection reduced to 52.8 epg (95% CI: 43.2–62.4 epg) in 2016 from 100.5 epg (95% CI: 88.7–112.3 epg) at baseline (*p* < 0.001). The prevalence of *Schistosoma haematobium* in the five MDA districts that had baseline prevalence data decreased to 2.2% (95% CI: 1.5–3.1%) in 2016 from 18.3% (95% CI: 16.3–20.5%) at baseline (*p* < 0.0001). Mean overall intensity of infection increased to 1.12 e/10 ml (95% CI: 0.55–0.1.70 e/10 ml) in 2016 compared to 0.47 e/10 ml (95% CI: 0.16–0.78 e/10 ml) in 2012 (*p* < 0.05) (no baseline data). No district was highly endemic in 2016 compared to three at baseline and there was no significant difference in prevalence or intensity of infection by sex for both species. This survey illustrated the significant progress made in controlling schistosomiasis in Sierra Leone. The fact that prevalence and intensity of infection showed an increase from the 2010 level suggested a detrimental effect of missing MDA due to the Ebola toward schistosomiasis control. The national program needs to continue the treatment and adopt a comprehensive approach including water, hygiene, and sanitation measures to achieve control and elimination of schistosomiasis.

## Background

Schistosomiasis was estimated to be the third leading cause of Disability Adjusted Life Years among neglected tropical diseases (NTDs) worldwide (1) and second after malaria in Sub-Saharan Africa accounting for 200,000 deaths annually (2–4). The two main human species in Africa are *Schistosoma mansoni* (causing intestinal schistosomiasis) and *Schistosoma haematobium* (causing urogenital schistosomiasis) that are transmitted via intermediate host snails *Biomphalaria* sp. and *Bulinus* sp., respectively (5–7).

Chronic intestinal schistosomiasis is manifested by hepatosplenomegaly (8), while urogenital schistosomiasis is associated with urinary obstructions, bladder cancer (9), and heightens the spread of HIV and its progress to AIDS (10, 11). Both can cause anemia due to inflammation, blood loss, and malnutrition and reduce life expectancy (12–15). The female schistosomes usual deposit eggs in the small venules of the portal and peri-vesical systems, but these may reach ectopic vessels draining the nervous system, causing seizures or neuropathies (16, 17) or those draining the reproductive organs causing fallopian tube obstruction, infertility or low birth weight (18, 19).

Schistosomiasis is one of five NTDs targeted with preventive chemotherapy for control/elimination. The World Health Organization (WHO) (20) strategy recommends that all school aged children (SAC) and “high risk” adults should receive praziquantel (PZQ) annually in communities highly endemic (prevalence ≥ 50%), biennially in communities moderately endemic (prevalence ≥ 10% and <50%) (21) and triennially for SACs only in low endemic communities (prevalence < 10%), (22). Adults are considered at high risk in occupations that expose them to snail infested fresh water. A reduction in the proportion of children with heavy intensity infections to less than 1% is anticipated after 2–3 rounds of effective MDA (22). To be effective each MDA should reach at least 75% of those at risk (22, 23).

Historical data from multiple studies have shown that both *S. mansoni* and *S. haematobium* were endemic in Sierra Leone (23–27). Malacological studies in the 1960s found that *Biomphalaria* and *Bulinus* sp were limited to 250 and 100 m above sea level respectively and not within 100 km of the Atlantic coast (28). In line with the snail distribution, the national mapping of *S. mansoni* and *S. haematobium* in 2008–2009 found high or moderate prevalence, in the seven, higher altitude, north-eastern districts and low prevalence or non-endemicity in the seven (low altitude) coastal districts (29–31). The mapping also showed that *S. mansoni* continues to become more prevalent than *S. haematobium* (32).

Annual/biennial MDA with PZQ started in six districts targeting only school-going children in 2009 and was scaled up to target all SACs (including non-school-going children) and at risk adults in all seven highly/moderately endemic districts in 2010. It was thought that transmission was not possible in the brackish waters of the coastal districts and that the few infected individuals who were found during baseline surveys were displaced from inland districts during the war and therefore MDA has not been conducted in these coastal districts to date.

In 2012, after 2–3 rounds of MDA with PZQ an impact assessment survey was conducted in the seven MDA districts, and showed an overall 65% reduction in the prevalence of *S. mansoni* infections (33). In 2016, after 3–6 rounds another assessment was conducted in both MDA and non-MDA districts. This paper presents the results and discusses the revised national schistosomiasis control strategy.

## Methods

### MDA, Coverage and Monitoring

In 2009, MDA with PZQ targeted school-going children only. Since 2010, MDA has targeted all SACs and at-risk adults except in 2014 when it was canceled due to the Ebola outbreak. The annual MDA follows social mobilization at district level, training or refresher training of distributors (strategy, dose poles, exclusion criteria, adverse events and their management), then social mobilization at community level. At-risk adults and non-enrolled SAC were targeted by community-based MDA and enrolled SAC were reached at school-based MDA. Owing to the potential adverse events of PZQ in heavily parasitized individuals the national policy has been that health workers (rather than volunteers or teachers) should be the distributors and that PZQ should only be given after a meal (34). Treatments were recorded on tally sheets which were summarized at the local health facility, then sent to the district NTD focal point for compilation and reporting to the national NTDP. Reported annual overall program coverage was between 78.5 and 96.3% from 2009 to 2015 as shown in Table S1. Effective coverage (≥75%) had been achieved since 2010 in each district each year except Koinadugu in 2010.

Independent monitoring of MDA coverage was conducted within a week of the MDA to assess performance and to validate the reported coverage, using the methodology described previously (35). In 2015, the independent monitoring preceding the impact survey sampled a total of 80 sites (40 randomly selected and 40 purposefully selected “hard to reach” sites) in the seven endemic districts. All eligible persons were interviewed at the household level and 30 males and 30 females were interviewed at community level. Independent monitoring was conducted in 2012, 2013, and 2015 as shown in Table S2. Most districts repeatedly achieved the minimum 75% coverage but coverage was <70% in Bo and Kailahun in 2013 and 2015.

### Survey Sites and Sample Sizes

The details of the baseline mapping surveys were described previously (29, 30). Briefly, in 2008, a total of 42 sites (4 sites/schools per district) were surveyed across the country and 100 children aged 5–16 years per school were randomly selected and examined (29). In 2009, in a complimentary baseline survey in the proposed seven MDA districts was performed: a total of 59 sites (schools) were surveyed in Bo, Bombali, Kailahun, Kenema, Kono, Tonkolili, and Koinadugu with 7–9 schools per district, and 30 children aged 9–14 years per school, examined ensuring equity by sex (30). In the 2012 impact assessment survey, a total of 33 sentinel sites were surveyed and 50 children aged 10–14 years (25 males, 25 females) were selected (31).

In 2016, a cross-sectional school-based survey was conducted in all 14 districts 6 months after the last MDA. A total of 72 sentinel sites were surveyed. Of these, 40 sites (schools) were from low, moderate and high prevalence communities identified at baseline from each of the seven MDA-districts, 5–6 sites per district, and additional 32 sites were from the seven non-MDA districts, 4–5 sites per district. To avoid repeated sampling that may raise awareness and bias the results, 38 sentinel sites were retained from those surveyed either at the baseline or in 2012 and 34 new sites were selected in 2016 [22 WHO]. Approximately 50 children aged 9–14 years were randomly selected per school.

Nationwide up to 38% of households were still reliant on an unimproved water source for drinking, of which 31% fetch water from a river or stream. In 10 districts, less than 10 percent of households had access to improved sanitation (36).

### Sample Collection and Examination

Upon arrival of the survey team at school, children were randomly selected according to the calculated sampling intervals and given a unique identification number, and their sex, and age were recorded. They were each given two plastic containers and asked to provide a fresh stool sample and a midday (10 a.m.−2 p.m.) terminal urine sample. Prior to the urine collection, about 15 min exercise was initiated by the survey team and teachers. All stool samples were examined (1 slide per sample) within 5 h of collection by the Kato-Katz method using a 41.7 mg thick fecal smear template and microscopy with a x10 lens (37). Number of eggs was counted, and results were expressed as number of eggs per gram of feces (epg). All positive and 10% of all negative slides were re-examined by senior technicians for quality assurance. All urine samples were examined using the filtration method (37). Ten milliliter of urine was filtered through a Twenty-micron polycarbonate membrane filter using a disposable syringe and a Swinnex filter holder. The filter was then examined with direct microscopy using a x10 objective lens (38). Number of eggs was counted and results were expressed as number of eggs per 10 ml of urine (e10 ml).

In 2008, Kato Katz was used for fresh stool examination in the field, but intensity was not calculated. In 2009, the stool samples were preserved using 10% formalin and transported to a laboratory in Freetown for examination. Fresh terminal urine samples were examined in field following manual centrifugation for 5 min and intensity data was not calculated.

### Data Analysis

Data were entered into Microsoft excel spreadsheet and exported for analysis using SPSS statistical software (IBM, version 23). The SPSS Complex Samples Crosstabs was used to calculate the prevalence and the Complex Samples Descriptive to calculate the mean egg counts with 95% confidence intervals (CI), taking into consideration the clustered nature of school children using districts as strata and schools as clusters. The arithmetic mean egg counts of infection from all those examined (including both positive and negative) was used in the analysis. Chi-squared test was used to compare prevalence and the Kruskal-Wallis test was used to compare mean egg counts between the studies and by sex. Intensity for *S. mansoni* was categorized as heavy (≥400 epg), moderate (100–399 epg), or light (1–99 epg) and for *S. haematobium* as heavy (≥50 e10 ml) or light (<50 e10 ml). GPS coordinates of the sites were collected and QGIS, Version 2.18.15 used to plot point prevalence distribution.

### Ethical Considerations

The surveys were part of the monitoring and evaluation activities of the national NTDP. Ethical approval for the survey was obtained from the Ethics and Scientific Review Committee of the Ministry of Health and Sanitation (MoHS), Sierra Leone. Community informed consent was obtained following discussion with District Medical Officers, head teachers, and community leaders. All communities were sensitized prior to sample collection and verbal consent was sought from parents. Written consent was obtained from the head teacher on behalf of the selected pupils who were also sensitized on the purpose of the activity. All children participated voluntarily, and the names were anonymized following sample collection. Those found positive were referred to the following MDA with PZQ.

## Results

### Prevalence of *S. mansoni* Infection

**Figure 1:**
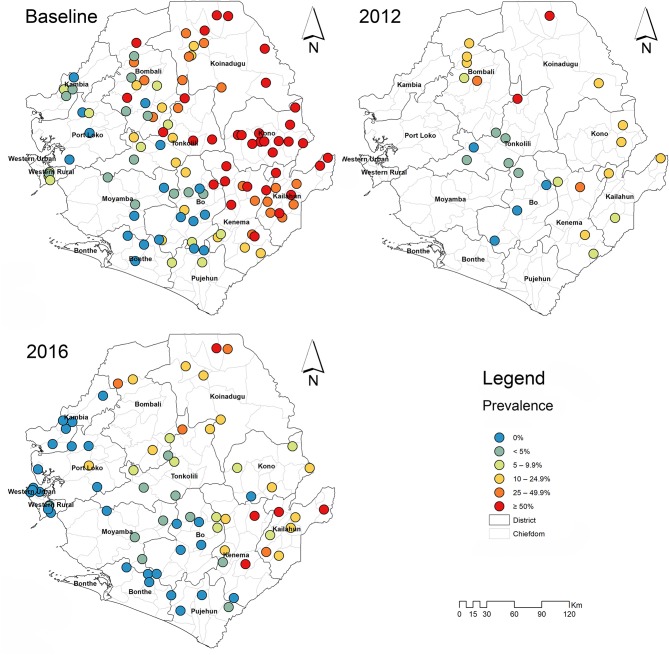
Survey sites and point prevalence maps for *S. mansoni* at baseline, 2012 and 2016 in Sierra Leone.

**Figure 2:**
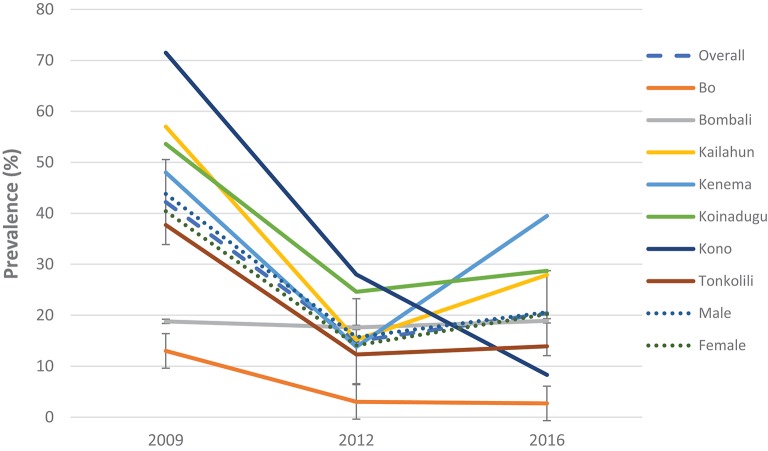
Prevalence of *S. mansoni* infection in 2009, 2012, and 2016 in the seven MDA districts, Sierra Leone.

In 2016, 1,980 stool samples from school age children (1,009 males and 971 females) and 1,382 urine samples (709 males, 673 females) were examined from 14 districts. The results are summarized in Table 1. The point prevalence and location of each survey site at three survey points are shown in Figure 1. The overall prevalence of *S. mansoni* was 11.4% (95% CI: 10.4–12.5%). It varied significantly from 0 to 1.6% in the seven non-MDA districts and 2.7–39.5% in the seven MDA districts. Kenema, Koinadugu, and Kailahun were among the districts with highest prevalence (39.5, 28.7, and 27.9%, respectively). Overall in all 14 districts (both MDA and non-MDA) there was no statistically significant difference in prevalence by sex: males 11.5% (95% CI: 10.1–13.0%) vs. females 11.3% (CI: 9.9–12.8%) (*p* > 0.05). This was similar when using data from the seven MDA districts: males 20.6% (95% CI: 18.2–23.3%) vs. females 20.2% (CI: 17.7–22.8%) (*p* > 0.05) as shown in Figure 2.

**Table 1:** Prevalence and mean egg counts of schistosome infections in school age children in 2016 in Sierra Leone.

	**No examined**	**No infected**	**Prevalence % (95% CI)**	**Heavily infected**	**Prevalence (%) of heavy infection(95% CI)**	**Mean egg counts (95% CI)^**a**^**
***S. mansoni***						
Overall	3632	414	11.4 (10.4–12.5)	74	2.0 (1.6–2.6)	29.1 (23.8–34.4)
Bo	294	8	2.7 (1.4–5.3)	0	0.0 (0.0–1.3)	1.9 (0.2–3.5)
Bombali	244	46	18.9 (14.4–24.2)	9	3.7 (2.0–6.9)	55.2 (20.4–89.9)
Kailahun	298	83	27.9 (23.1–33.2)	11	3.7 (2.1–6.5)	47.1 (27.7–66.5)
Kenema	301	119	39.5 (34.2–45.2)	30	10.0 (7.1–13.9)	143.3 (102.5–184.1)
Koinadugu	300	86	28.7 (23.8–34.0)	14	4.7 (2.8–7.7)	65.0 (42.1–87.8)
Kono	242	20	8.3 (5.4–12.4)	1	0.4 (0.1–2.3)	7.5 (0.0–15.4)
Tonkolili	301	42	14.0 (10.5–18.3)	8	2.7 (1.4–5.2)	39.8 (18.6–61.0)
Port Loko	249	1	0.4 (0.1–2.2)	0	0.0 (0.0–1.5)	0.6 (0.0–1.7)
Kambia	250	1	0.4 (0.1–2.2)	0	0.0 (0.0–1.5)	0.6 (0.0–1.1)
Bonthe	251	0	0.0 (0.0–1.5)	0	0.0 (0.0–1.5)	0.0 (0.0–0.0)
Moyamba	252	4	1.6 (0.6–4.0)	0	0.0 (0.0–1.5)	1.2 (0.0–3.1)
Pujehun	252	3	1.2 (0.4–3.4)	0	0.0 (0.0–1.5)	0.4 (0.0–0.8)
RWA	201	1	0.5 (0.1–2.8)	1	0.5 (0.1–2.8)	3.3 (0.0–9.9)
UWA	197	0	0.0 (0.0–1.9)	0	0.0 (0.0–1.9)	0.0 (0.0–0.0)
By Sex						
Male	1844	212	11.5 (10.1–13.0)	42	2.3 (1.7–3.1)	25.9 (20.0–31.8)
Female	1788	202	11.3 (9.9–12.8)	32	1.8 (1.3–2.5)	32.4 (23.5–41.3)
***S. haematobium***						
Overall	2983	53	1.8 (1.4–2.3)	16	0.5 (0.3–0.9)	0.8 (0.4–1.2)
Bo	294	8	2.7 (1.4–5.3)	1	0.3 (0.1–1.9)	0.5 (0.0–1.0)
Bombali	244	2	0.8 (0.2–2.9)	2	0.8 (0.2–2.9)	1.5 (0.0–3.6)
Kailahun	298	1	0.3 (0.1–1.9)	1	0.3 (0.1–1.9)	0.2 (0.0–0.5)
Kenema	301	3	1.0 (0.3–2.9)	0	0.0 (0.0–1.3)	0.1 (0.0–0.3)
Koinadugu	300	21	7.0 (4.6–10.5)	6	2.0 (0.9–4.3)	2.5 (0.9–4.1)
Kono	242	3	1.2 (0.4–3.6)	1	0.4 (0.1–2.3)	0.5 (0.0–1.5)
Tonkolili	301	14	4.7 (2.8–7.7)	4	1.3 (0.5–3.4)	2.5 (0.0–5.6)
Port Loko	249	0	0.0 (0.0–1.5)	0	0.0 (0.0–1.5)	0.0 (0.0–0.0)
Kambia	250	1	0.4 (0.1–2.2)	1	0.4 (0.1–2.2)	0.2 (0.0–0.6)
Bonthe	–		–	–	–	–
Moyamba	252	0	0.0 (0.0–1.5)	0	0.0 (0.0–1.5)	0.0 (0.0–0.0)
Pujehun	252	0	0.0 (0.0–1.5)	0	0.0 (0.0–1.5)	0.0 (0.0–0.0)
RWA	–	–	–	–	–	–
UWA	–	–	–	–	–	–
By Sex						
Male	1530	30	2.0 (1.4–2.8)	7	0.5 (0.2–0.9)	0.5 (0.2–0.8)
Female	1453	23	1.6 (1.1–2.4)	9	0.6 (0.3–1.2)	1.0 (0.3–1.7)

a *Mean egg counts are expressed as epg for S. mansoni and e10 ml for S. haematobium*.

**Figure 3:**
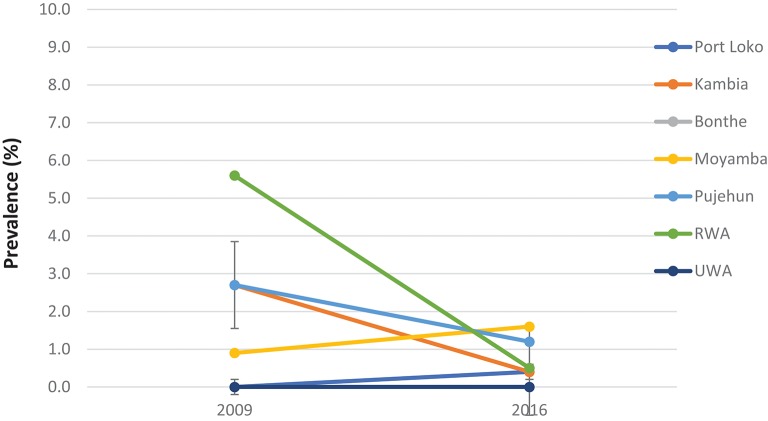
Prevalence of *S. mansoni* infection in the seven non-MDA districts in 2009 and 2016, Sierra Leone.

Compared with the baseline, *S. mansoni* prevalence in the seven MDA districts that were highly/moderately endemic, there was a significant reduction in overall prevalence from 42.2% (95% CI: 39.8–44.5%) at baseline to 20.4% (95% CI: 18.7–22.3%) in 2016 which was however a slight increase from 14.9% (95% CI: 13.2–16.7%) in 2012 (*p* < 0.001) (Figure 2). In the seven non-MDA districts *S. mansoni* prevalence remained very low in 2016 (<2%), with Kambia, Pujehun, and RWA showing even lower prevalence than the baseline (*p* < 0.01) (Figure 3).

Among the seven MDA districts, Bombali did not show significant change in prevalence over three surveys (*p* > 0.05); the other six districts showed significant decrease in 2012 from the baseline (*p* < 0.01); but in 2016 Kailahun, Kenema, and Koinadugu showed significant rebound in prevalence from the 2012 level (*p* < 0.01) while Bo and Tonkolili stayed flat from 2012 and only Kono continued to decrease (Figure 2). Both males and females showed similar trend in prevalence over three survey points, with decreasing from 43.8% (95% CI: 40.5–47.1%) at baseline to 15.7% (95% CI: 13.3–18.4%) in 2012 then increasing to 20.6% (95% CI: 18.2–23.3) in 2016 in males and decreasing from 40.4% (37.1–43.8) at baseline to 14.1% (95% CI: 11.8–16.7%) in 2012 then increasing to 20.2% (CI: 17.7–22.8%) in 2016 in females (*p* < 0.05 each) as shown in Figure 2.

### Mean Egg Counts of *S. mansoni* Infection

**Figure 4:**
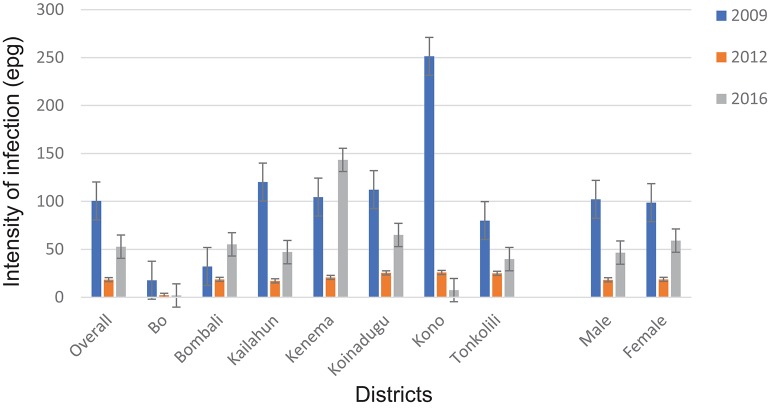
Arithmetic mean egg counts of *S. mansoni* infection in 2009, 2012 and 2016 in the seven MDA districts, Sierra Leone.

The overall mean egg count in 2016 was 29.1 epg (95% CI: 23.8–34.4 epg) in 14 districts as shown in Table 1. In the seven MDA districts, the mean egg counts varied by district between 1.9 epg (95% CI: 0.2–3.5 epg) in Bo and 143.3 epg (95% CI: 102.5–184.1 epg) in Kenema. The mean egg count was 46.6 epg (95% CI: 36.0–57.2 epg) in males and 59.1 epg (95% CI: 42.9–75.3 epg) in females in the seven MDA districts (Figure 4) (*p* > 0.05). The few infections in the seven non-MDA districts were very light and no comparison was made due to the lack of egg count data at the baseline.

Compared with the baseline (2009), among the seven MDA districts, there was a significant reduction in overall mean egg count from 100.5 epg (95% CI: 88.7–112.3 epg) to 52.8 epg (95% CI: 43.2–62.4 epg) in 2016 (*p* = 0.001) (Figure 4). Four of the seven districts (Bo, Kailahun, Koinadugu, and Kono) also showed a significant reduction in mean egg counts in 2016 from the baseline (*p* < 0.05 each). Overall there was a significant increase in 2016 from 18.4 epg (95% CI:14.7–22.1 epg) in 2012 (*p* < 0.001) (Figure 4). Four districts (Bombali, Kailahun, Kenema, and Koinadugu) showed an increase from 2012 and Bombali and Kenema even had an increase from the baseline level (Figure 4). Only Kono showed continuous reduction over three survey points. Similar to the prevalence above, there was significant reduction in mean egg counts in both males and females (*p* = 0.001 each) from the baseline, but a significant increase from 2012 (*p* < 0.01).

**Figure 5:**
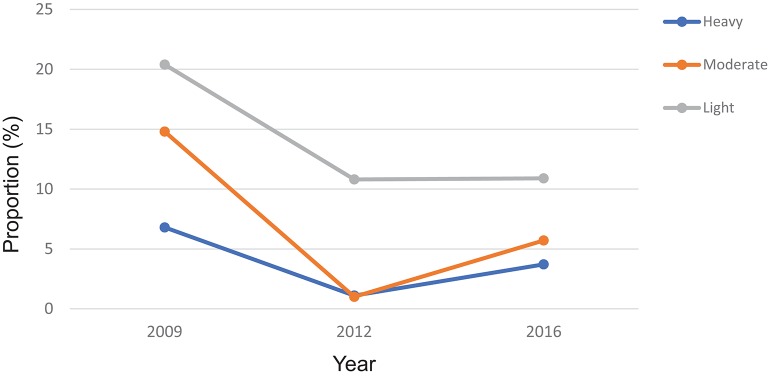
Proportion of heavy, moderate and light intensity of *S. mansoni* infection in 2009, 2012, and 2016 in Sierra Leone.

According to the WHO infection categories, there was on average 2.0% (95% CI: 1.6–2.6%) of heavy infections across the 14 districts and 5 districts (Bombali, Kailahun, Kenema, Koinadugu, and Tonkolili) had heavy infections between 2.7 and 10.0% (Table 1). Among the seven MDA districts, there were 3.7% (95% CI: 2.9–4.6%) heavy infections and 5.9% (95% CI: 4.9–7.0%) moderate infections in 2016, which were significantly reduced from 6.8 to 14.8% at the baseline (*p* < 0.0001), but significantly increased from 1.1 and 1.0% in 2012 (*p* < 0.001) (Figure 5).

### Prevalence and **M**ean Egg Counts of *S. haematobium* Infection

There was a low level of *S. haematobium* infection with an overall prevalence of 1.8% (95% CI: 1.4–2.3%), but mainly in the seven MDA districts (Table 1). Among the seven MDA districts, *S. haematobium* prevalence varied from 0.3 to 7.0% and the mean egg counts varied from 0.1 to 2.5 e10 ml. There was on average 0.5% (95% CI: 0.3–0.9%) heavy infection, with only two districts above 1% (Koinadugu 2.0% and Tonkolili 1.3%).

Only five districts had baseline data and therefore the data from these five districts were used for comparison between the surveys. There was a significant reduction in overall *S. haematobium* prevalence from 18.3 (95% CI: 16.3–20.5%) at baseline to 2.2% (95% CI: 1.5–3.1%) in 2016 (*p* < 0.001) as shown in Table 2. There was a significant increase in prevalence in Tonkolili district from zero to 4.7% (*p* < 0.05). No district had moderate or high prevalence in 2016 compared to 2 districts (Bo and Kono) with moderate prevalence at baseline. The mean intensity was 1.1 e10 ml (95% CI: 0.6–1.7 e10 ml) in 2016 (no baseline intensity data available).

**Table 2:** Prevalence and mean egg counts of *S. haematobium* infection in 2009, 2012 and 2016 in five districts.

	**2009**		**2012**		**2016**	
	***n***	**% (95% CI)**	***n***	**% (95% CI)**	***n***	**% (95% CI)**
**Overall**	**1,338**	**18.3 (16.3–20.5)**	**1,230**	**3.3 (2.4–4.4)**	**1,382**	**2.2 (1.5–3.1)**^**a**^
**BY DISTRICT**						
Bo	675	24.6 (21.4–28.1)	294	8.8 (5.9–12.7)	294	2.7 (1.2–5.3)^b^, ^d^
Bombali	261	5.7 (3.3–9.3)	239	2.5 (0.9–5.4)	244	0.8 (0.1–2.9)
Kenema	60	0.0 (0.0–6.0)	244	0.8 (0.1–2.9)	301	1.0 (0.3–3.1)
Kono	253	25.3 (20.1–31.1)	151	2.0 (0.4–5.7)	242	1.2 (0.3–3.6)^b^
Tonkolili	89	0.0 (0.0–4.1)	302	1.0 (0.3–3.1)	301	4.7 (2.7–7.9)^c^
**By Sex**						
Male	747	18.5 (15.8–21.5)	617	3.2 (2.0–5.1)	709	2.7 (1.7–4.2)^b^
Female	591	18.1 (15.1–21.5)	613	3.3 (2.1–5.1)	673	1.6 (0.9–3.0)^b^
**Overall**				**Eggs/10 ml**		**Eggs/10 ml**
**Mean intensity**			**1,230**	**0.5 (0.1–0.9)**		**1.1 (0.6–1.7)**^**e**^
Bo			294	1.8 (0.3–3.4)		0.5 (0.0–1.1)
Bombali			239	0.2 (0.0–0.5)		1.5 (0.0–3.6)
Kenema			244	0.0 (0.0–0.0)		0.1 (0.0–0.3)
Kono			151	0.1 (0.0–0.3)		0.5 (0.0–1.5)
Tonkolili			302	0.1 (0.0–0.1)		2.5 (0.0–5.3)
**BY SEX**						
Male			617	0.7 (0.0–1.4)		0.7 (0.1–1.2)
Female			613	0.3 (0.1–0.6)		1.5 (0.1–2.9)
**PROPORTION OF HEAVY AND LIGHT INTENSITY**						
Heavy	-	-	1,230	0.2%	1,382	0.4%
Light	-	-	1,230	3.4%	1,382	1.6 %

a Overall significant decrease in prevalence from 2009 to 2016 (p < 0.0001),

b Significant decrease (p < 0.05) from baseline;

c Significant increase (p < 0.05) from baseline;

d Significant reduction (p < 0.01) from 2012,

e *Overall significant increase in intensity from 2012 to 2016 (p < 0.0001)*.

Overall, or by sex there was no significant change in prevalence compared with 2012. One district (Bo) showed significant reduction in prevalence (*p* < 0.005). The overall mean intensity increased from 0.5 eggs/10 ml (95% CI: 0.1–0.9) in 2012 to 1.1 eggs/10 ml (95% CI: 0.6–1.7) in 2016 (*p* < 0.05).

## Discussion

Two of the specific objectives of the 2010–2015 NTD Master Plan for schistosomiasis control in Sierra Leone were (1) to treat at least 80% of SAC and at-risk adult population in endemic communities with PZQ and (2) to reduce prevalence among SAC in all districts to less than 10% (39). Reported MDA coverage has been above the WHO recommended coverage since 2010 according to the NTDP reports. The sentinel site survey in 2016 showed that the overall prevalence and intensity of *S. mansoni* infection in Sierra Leone was 11.4% and 29.1 epg, respectively, and the overall prevalence and intensity of *S. haematobium* infection was 1.8% and 0.8 e10 ml respectively.

In line with the mapping results (30–32), the seven coastal districts still had very low prevalence of schistosomiasis and in some districts there was a reduction in prevalence without conducting MDA. The results justified the national decision to not conduct MDA for morbidity control in these seven districts in line with the malacological data at this stage (40). In the seven MDA districts, there was a 51.7% reduction in prevalence for *S. mansoni* from the baseline. For *S. haematobium* in the five districts that had baseline data there was an 88.0% reduction in prevalence. Two of the seven MDA districts reached the national target of reducing prevalence in SACs to below 10%, while the other five MDA districts still had 13.9–39.5% *S. mansoni* prevalence with heavy infections at 2.7–10.0% after six rounds of MDA over 7 years.

Despite the success achieved in the overall MDA coverage, there had been challenges in reaching some people. In certain places, the actual coverage may not have been as high as reported. The post-event surveys by independent monitors found MDA coverage had been lower than that reported by the NTDP especially in two districts (Bo and Kailahun). Possible explanations may include: (1) underestimates of target populations as the projected growth rate of 2.4% in the national census in 2004 was amended to 3.2% in the national census in 2015; (2) the biennial phases of MDA in chiefdoms in Bo where SAC move considerably to attend educational resources away from home; (3) in Kailahun which borders both Guinea and Liberia the considerable cross-border migration to access PZQ during MDA in Sierra Leone; and (4) limited resources for health workers to return to communities for missed eligible persons in remote communities. Independent monitoring also identified common reasons for noncompliance in taking PZQ which included experience of previous adverse events either by self, a close friend or relative such as nausea, dizziness, diarrhea, vomiting, and headache.

Prevalence and intensity in 2016 did not show further reduction after 2012 and in three border districts there was an increase: Kailahun, Kenema, and Koinadugu. One of the factors may have been due to the fact that only one round of MDA occurred between 2012 and 2015 in moderately endemic communities and heavily infected communities also missed one round of MDA due to the Ebola emergency in 2014. In Kenema which had the highest prevalence in 2016, one of three original sentinel sites (Lower Bambara) had the highest prevalence and intensity at baseline (96%, 130.9 epg), suggesting a high level of local transmission in certain areas. The increase at one site in Port Loko and of *S. haematobium* infection in Tonkolili could be attributed to the rapid employment-seeking migration from more highly endemic communities as a result of the opening a railway serving new iron-ore mines in 2012 and/or alterations to snail populations from irrigation systems built to sustain a large sugar cane plantation and a dam for hydroelectricity production. In addition, ~50% of the sentinel sites from the baseline were changed in the 2016 survey, with purposefully selected sites from other areas where no baseline data were available. All these may have contributed to the increases in both prevalence and intensity of *S. mansoni* seen in this survey in 2016 from 2012.

By comparison, in West Africa, after 5 years of MDA in Mali there was no significant reduction in prevalence of *S. mansoni* from baseline (41). In Burkina Faso, after 5 years of MDA one third of sentinel sites still had high prevalence of schistosomiasis (42) and after 10 years of MDA the prevalence of *S. haematobium* remained high (>18%) in 3 of the 11 MDA-regions (43). In East Africa, biannual MDA in Zanzibar focusing on schistosomiasis elimination has been performed since 2012 but *S. haematobium* prevalence remained high in hotspots. These hotspots were characterized by fewer reliably working taps and shorter distances between schools and Human Water Contact Sites containing *Bulinus* globosus (44).

In Sierra Leone a large proportion of households and schools across the country still lack access to improved water (protected wells or springs) and sanitation (flush toilets, pit latrines). In 2015, an estimated 50% of rural households were still reliant on unimproved sources (45) for drinking water (unprotected well or spring, rain waters, rivers, streams, lakes, ponds) with one MDA-district Tonkolili being amongst the highest: 56% (36). Open defecation was still practiced by many rural households Kailahun being amongst the highest: 48% (36). In the long term a reduction of the transmission of schistosomiasis will require effective sanitation and improved water supplies to reduce the need to use and pollute natural river water for domestic purposes (46). Health education, and behavior change communication within communities are required to ensure their appropriate usage (47). The Government of Sierra Leone recently adopted sustainable development goal (SDG) targets of improving water quality and reducing the proportion of untreated water by half by 2030 (48). Novel means of reducing snail populations by introducing prawns might also be worthy of investigation (49, 50).

In line with the WHO recommendations (23) and based on the current survey results, it is recommended that the NTDP should (1) continue and intensify annual MDA in Bombali, Kailahun, Kenema, Koinadugu, and Tonkolili districts and biennial MDA in Bo and Kono districts, (2) further coordinate the schistosomiasis control effort with the WASH activities to maximize the program impact, and (3) further investigate factors hindering the program impact and make necessary adjustment in MDA strategy.

There were certain limitations in the study. Firstly, there were differences in the sample sizes and sampling methods used in different surveys in different years. This made the comparison of the results between years difficult. Secondly, the surveys were conducted in school age children, and the situation in other at-risk age groups is not known. Future surveys should consider sampling from other at-risk age groups. Other potential cofounding factors that may have influenced these results relate to personal hygiene, access to safe drinking water and improved sanitation. There has been considerable effort to improve the practice of hand-washing with soap and the safe disposal of human feces before which was intensified during the Ebola emergency but overall the situation remains poor and continues to need attention if transmission is to be reduced and improvements sustained.

In conclusion, by 2016 Sierra Leone had made major progress in reducing prevalence and intensity of schistosomiasis infection, compared with the baseline in 2008/9. However, the Ebola outbreak in 2014 and other factors have hindered the progress of schistosomiasis control. The NTDP should continue MDAs and endevour to reach all at risk communities especially in the three border districts whilst coordinating with WASH partners and their activities to reduce transmission.

## Consent for Publication

Although consent was obtained from participants before the study, data collection was conducted such that participants remained anonymous during data entry and analysis. No individuals' identity can be revealed upon publication.

## Data Availability Statement

All data generated or analyzed during this study are included in this published article. The dataset analyzed is available from the corresponding author on reasonable request and can be made available with permission from the MoHS Sierra Leone.

## Ethics Statement

The impact assessment was part of the routine monitoring and evaluation activities of the national NTDP. Ethical approval was obtained from the Ethics and Scientific Review Committee of the MoHS. Community informed consent was obtained following discussion with District Medical Officers, Chiefdom school inspectors, head teachers and community-teachers' associations. All communities included in the survey were sensitized by representatives of the district NTDP and the survey teams upon arrival prior to sample collection. Verbal consent was sought from parents during these meetings. A written consent was obtained from the school head teacher and the village head on behalf of the pupils recruited. Children enlisted were also sensitized on the purpose of the activity prior to sample collection. Participation was voluntary. All data generated or analyzed during this study are included in this published article and its supplementary files.

## Author Contributions

YB and AC managed and supervised the national NTDP. JP, MB, MS, AV, and MH planned and coordinated the survey. JP, MB, AT, and SS collected the field data and performed the laboratory investigations. MB and JP performed the data analysis. JP wrote the first draft of the manuscript. MH and YZ revised the paper. All authors approved the final manuscript.

### Conflict of Interest Statement

The authors declare that the research was conducted in the absence of any commercial or financial relationships that could be construed as a potential conflict of interest.

## Abbreviations

DHMT: District Health Management Team
MoHS: Ministry of Health and Sanitation
NTD: Neglected tropical diseases
NTDP: Neglected Tropical Disease Program
MDA: Mass Drug Administration
USAID: United States Agency for International Development
WHO: World Health Organization.
