# Supplementary material for: Schistosomiasis in School Age Children in Sierra Leone After 6 Years of Mass Drug Administration With Praziquantel
Source: Front Public Health. 2019 Feb 12;7:1. doi: 10.3389/fpubh.2019.00001 (PMC6379326; doi:10.3389/fpubh.2019.00001)
Supplement: Supplementary file 1 [file Table_1.DOCX]

**Table S1. Annual reported coverage of mass drug administration by district in Sierra Leone^1,2^**

|  | **2009** | | | **2010** | | | **2011** | | | **2012** | | | **2013** | | | **2015** | | |
| --- | --- | --- | --- | --- | --- | --- | --- | --- | --- | --- | --- | --- | --- | --- | --- | --- | --- | --- |
| **District** | **Eligible** | **Treated** | **%** | **Eligible** | **Treated** | **%** | **Eligible** | **Treated** | **%** | **Eligible** | **Treated** | **%** | **Eligible** | **Treated** | **%** | **Eligible** | **Treated** | **%** |
| Kailahun | 108,375 | 84,118 | 78 | 97,556 | 84,444 | 87 | 97,556 | 84,030 | 86 | 121,427 | 82,213 | 68 | 49,400 | 30,968 | 63 | 130,382 | 102,408 | 79 |
| Kenema | 152,533 | 151,203 | 99 | 102,443 | 89,764 | 88 | 100,439 | 84,868 | 84 | 164,783 | 84,891 | 52 | 63,158 | 57,853 | 92 | 173,316 | 144,753 | 84 |
| Kono | 121,561 | 97,279 | 80 | 124,550 | 68,369 | 55 | 84,468 | 72,917 | 86 | 137,095 | 71,371 | 52 | 140,385 | 105,175 | 75 | 147,210 | 121,315 | 82 |
| Bombali |  |  |  | 32,394 | 29,756 | 92 | 4,562 | 3,939 | 86 | 6,114 | 4,568 | 75 | 134,931 | 108,276 | 80 | 61,261 | 50,553 | 83 |
| Koinadugu | 77,941 | 55,725 | 71 | 84,987 | 50,631 | 60 | 59,121 | 50,631 | 86 | 94,572 | 49,118 | 52 | 36,605 | 32,720 | 89 | 108,859 | 89,003 | 82 |
| Tonkolili | 100,967 | 64,355 | 64 | 135,069 | 118,943 | 88 | 50,122 | 43,282 | 86 | 46,827 | 43,147 | 92 | 103,695 | 70,532 | 68 | 87,906 | 73,059 | 83 |
| Bo | 153,946 | 110,300 | 72 | 113,500 | 98,196 | 87 | 75,679 | 63,545 | 84 | 50,742 | 63,812 | 126 | 93,483 | 87,647 | 94 | 144,804 | 115,279 | 80 |
| **Total** | 715,323 | 562,980 | 79 | 90,499 | 540,103 | 78 | 471,947 | 403,212 | 85 | 621,560 | 99,120 | 64 | 621,657 | 493,171 | 79 | 853,737 | 696,370 | 82 |

Note: ^1^ there was no MDA in 2014 due to the Ebola outbreak. ^2^ Coverage data for 2009-2011 have been published previously [34]. High coverage in Bo in 2012 was partly due to school-aged children being sent into Kakua chiefdom from neighboring chiefdoms/districts as school there are more numerous and generally of good standard.
