# Supplementary material for: Schistosomiasis in School Age Children in Sierra Leone After 6 Years of Mass Drug Administration With Praziquantel
Source: Front Public Health. 2019 Feb 12;7:1. doi: 10.3389/fpubh.2019.00001 (PMC6379326; doi:10.3389/fpubh.2019.00001)
Supplement: Supplementary file 2 [file Table_2.DOCX]

**Table S2. MDA coverage by independent monitoring surveys in 2012, 2013 and 2015 in Sierra Leone**

|  | **2012** | | | | **2013** | | | | **2015** | | | |
| --- | --- | --- | --- | --- | --- | --- | --- | --- | --- | --- | --- | --- |
| **District** | **No of persons interviewed** | **No of persons recalling having taken PZQ** | **%** | **No of persons interviewed** | | **No of persons recalling having taken PZQ** | **%** | **No of persons interviewed** | | **No of persons recalling having taken PZQ** | **%** |  |
| Bo | 254 | 214 | 84.3 | 766 | | 457 | 59.7 | 2024 | | 1388 | 68.6 |  |
| Bombali | 61 | 45 | 73.8 | 738 | | 570 | 77.2 | 1183 | | 1027 | 86.8 |  |
| Kailahun | 846 | 651 | 77.0 | 334 | | 206 | 61.7 | 1935 | | 1132 | 58.5 |  |
| Kenema | 948 | 726 | 76.6 | 480 | | 401 | 83.5 | 2770 | | 2002 | 72.3 |  |
| Koinadugu | 719 | 644 | 89.6 | 294 | | 246 | 83.7 | 1780 | | 1338 | 75.2 |  |
| Kono | 2,037 | 1,678 | 82.4 | 958 | | 729 | 76.1 | 2,063 | | 1,703 | 82.5 |  |
| Tonkolili | 259 | 183 | 70.7 | 710 | | 530 | 74.6 | 1335 | | 1060 | 79.4 |  |
| **Overall** | **5,124** | **4141** | **80.8** | **4,280** | | **3,139** | **73.3** | **13,090** | | **9,650** | **73.7** |  |
